# Convergent Adaptation of Multiple Herbicide Resistance to Auxin Mimics and ALS- and EPSPS-Inhibitors in *Brassica rapa* from North and South America

**DOI:** 10.3390/plants12112119

**Published:** 2023-05-26

**Authors:** José Alfredo Dominguez-Valenzuela, Candelario Palma-Bautista, José G. Vazquez-Garcia, Marcos Yanniccari, Ramón Gigón, Ricardo Alcántara-de la Cruz, Rafael De Prado, João Portugal

**Affiliations:** 1 Department of Agricultural Parasitology, Chapingo Autonomous University, Texcoco 56230, Mexico; jose_dv001@yahoo.com.mx; 2 Department Agroforestry, Biochemistry and Molecular Biology, University of Cordoba, 14014 Cordoba, Spain; z82vagaj@uco.es (J.G.V.-G.); qe1pramr@uco.es (R.D.P.); 3 Chacra Experimental Integrada Barrow (MDA-INTA), National Scientific and Technical Research Council (CONICET), Faculty of Agronomy, National University of La Pampa, Santa Rosa L6300, Argentina; 4 Private Consultant in Weed Control, Buenos Aires C1033, Argentina; gigonramon@gmail.com; 5 Departamento de Química, Universidade Federal de São Carlos, São Carlos 13565-905, Brazil; ricardo.cruz@ufscar.br; 6 Biosciences Department, Polytechnic Institute of Beja, 7800-000 Beja, Portugal; jportugal@ipbeja.pt; 7 VALORIZA-Research Centre for Endogenous Resource Valorization, Polytechnic Institute of Portalegre, 7300-555 Portalegre, Portugal

**Keywords:** ALS enzyme activity, ethylene production, herbicide tolerant crops, oilseed rape, resistant gene flow, shikimic acid accumulation

## Abstract

Herbicide-resistant weeds have been identified and recorded on every continent where croplands are available. Despite the diversity of weed communities, it is of interest how selection has led to the same consequences in distant regions. *Brassica rapa* is a widespread naturalized weed that is found throughout temperate North and South America, and it is a frequent weed among winter cereal crops in Argentina and in Mexico. Broadleaf weed control is based on glyphosate that is used prior to sowing and sulfonylureas or mimic auxin herbicides that are used once the weeds have already emerged. This study was aimed at determining whether a convergent phenotypic adaptation to multiple herbicides had occurred in *B. rapa* populations from Mexico and Argentina by comparing the herbicide sensitivity to inhibitors of the acetolactate synthase (ALS), 5-enolpyruvylshikimate-3-phosphate (EPSPS), and auxin mimics. Five *B. rapa* populations were analyzed from seeds collected in wheat fields in Argentina (Ar1 and Ar2) and barley fields in Mexico (Mx1, Mx2 and MxS). Mx1, Mx2, and Ar1 populations presented multiple resistance to ALS- and EPSPS-inhibitors and to auxin mimics (2,4-D, MCPA, and fluroxypyr), while the Ar2 population showed resistance only to ALS-inhibitors and glyphosate. Resistance factors ranged from 947 to 4069 for tribenuron-methyl, from 1.5 to 9.4 for 2,4-D, and from 2.7 to 42 for glyphosate. These were consistent with ALS activity, ethylene production, and shikimate accumulation analyses in response to tribenuron-methyl, 2,4-D, and glyphosate, respectively. These results fully support the evolution of the multiple- and cross-herbicide resistance to glyphosate, ALS-inhibitors, and auxinic herbicides in *B. rapa* populations from Mexico and Argentina.

## 1. Introduction

Weeds evolve and adapt to the environment in response to management practices [1,2]. Herbicide-resistant populations are considered adaptative evolutionary processes that take place across the agricultural landscape [3]. In every continent where croplands are available, the occurrence of herbicide-resistant weeds has been recorded [4]. While the spontaneous plant communities and agricultural forms of production vary throughout the world, chemical control is the primary tool for weed management in most countries [5]. Glyphosate is the most widely applied herbicide worldwide and some of the most intensive uses of glyphosate occur in North and South America in reduced-tillage cropping systems and on glyphosate-resistant crops [6,7].

The overreliance on glyphosate across vast regions with a low diversity of control strategies explains the increase in glyphosate-resistant cases [4,8]. Despite the low to medium risk of glyphosate resistance evolution [9,10], the widespread and repeated use of this herbicide has led to a selection of weeds that have become resistant to glyphosate [11]. The challenge of controlling herbicide-resistant weeds increases when the evolution of multiple resistance emerges in response to the repetitive use of herbicides with the same site of action to manage the weeds’ resistance to other sites of action [12].

Additionally, the role of herbicide selection in the evolution of resistance is conditioned by genetic variations within weed populations [13]. The genetic variations among a population are defined by the rate of spontaneous new mutations, gene flow processes, genetic recombinations, and genetic drifts, where herbicide selection can fulfill its job. The intraspecific variability of herbicide sensitivity may be naturally occurring or induced by genetic engineering. In the first case, most cases of resistance have been reported in weed populations, and herbicide-resistant transgenic crops make up the second case [14,15]. Interestingly, the evolution of the herbicide-resistant *Brassica rapa* in Argentina occurred through gene flow and introgression between feral populations of glyphosate-resistant transgenic *B. napus* (oilseed rape), imidazolinone-tolerant non-transgenic cultivars, and wild *B. rapa*, where the persistence and spread of resistance were facilitated by the high selection pressure imposed by glyphosate and acetolactate synthase (ALS)-inhibiting herbicides [16,17].

More than 200 weed species have evolved to become resistant to herbicides worldwide, but only 3 botanical families (Poaceae, Asteraceae, and Brassicaceae) make up more than half of these species [4,18]. Despite the diversity of weed communities, it is of interest how selection has led to the same consequences in distant regions. Thus, the convergent phenotypic evolution of herbicide resistance has been linked to the selection pressure imposed by herbicides in different croplands [5,19].

*Brassica rapa* is a widespread naturalized weed found throughout temperate North and South America, and it is a frequent weed found among winter cereal crops. *Brassica rapa* is a very common weed among cereals grown in temperate climates and they are widely distributed in Mexico, especially in the High Valleys, as well as in the Argentinean Pampas [17,20,21]. In these Argentinean and Mexican systems, broadleaf weed control is based on glyphosate use in pre-sowing and on sulfonylureas or auxin mimic herbicides, which are used once the weeds have already emerged. Under different environments in both countries, the selection of herbicide-resistant *B. rapa* plants can take place; however, is the coevolution of multiple herbicide-resistant *B. rapa* populations in Argentina and Mexico possible? Can different patterns of use of herbicides produce the same phenotypic selection using different *B. rapa* populations? The study aimed to determine whether a convergent phenotypic adaptation process has occurred in *B. rapa* populations from Mexico and Argentina by comparing the populations’ herbicide sensitivities to ALS, 5-enolpyruvylshikimate-3-phosphate (EPSPS), and auxin mimics.

## 2. Results

### 2.1. Herbicide-Sensitivity: Cross- and Multiple-Herbicide Resistance

The Argentinean and Mexican *B. rapa* populations were susceptible to atrazine, bromoxynil, glufosinate, and mesotrione (inhibitors of the photosystem I–PSI, photosystem II–PSII, glutamine synthetase–GS, and 4-hydroxyphenylpyruvate dioxygenase–HPPD, respectively). In contrast, all susceptible and resistant *B. rapa* populations showed a natural tolerance to the auxin mimics clopyralid and dicamba and presented a high production of fresh mass and survival rates of 100%. Mx1, Mx2, and Ar1 populations showed resistance to ALS inhibitors (tribenuron-methyl, florasulam, imazamox, and iodosulfuron + mesosulfuron), auxin mimics (2,4-D, MCPA, and fluroxypyr), and glyphosate, while the Ar2 population showed resistance only to ALS and EPSPS inhibitors (Table 1).

### 2.2. Dose–Response to TM, 2,4-D, and Glyphosate

The MxS *B. rapa* population was very sensitive to TM, 2,4-D, and glyphosate. Based on GR_50_ and LD_50_, the resistant populations survived at TM doses that were 13 to 110 times the field dose (20 g ai ha^−1^) and the RFs ranged from 947 to 4069 in relation to the MxS population. Among the resistant populations, Mx1 was the most sensitive to TM and Ar2 was the most resistant to TM; there were no differences between Mx2 and Ar1.

In response to 2,4-D, GR_50_, and LD_50_ values for Mx1, Mx2, and Ar1 populations confirmed their resistance to this herbicide with RFs at around 5.0 to 9.4. For MxS and Ar2, the GR_50_ and LD_50_ values were similar and both populations were controlled at rates lower than the field dose (600 g ai ha^−1^).

With respect to glyphosate, the Argentinean populations showed a higher resistance level (GR_50_ > 2300 g ae ha^−1^) compared to the Mexican populations. Although Ar2 was more sensitive to glyphosate than Ar1, both showed RFs ≥ 20. LD_50_ values for Mx1 and Mx2 were higher than MxS, but these parameters were lower than the field dose (793 and 600, respectively, versus 960 g ae ha^−1^) and the RFs were at around 2.7–3.5 (Table 2).

### 2.3. ALS Enzyme Activity in Response to TM

**Figure 1:**
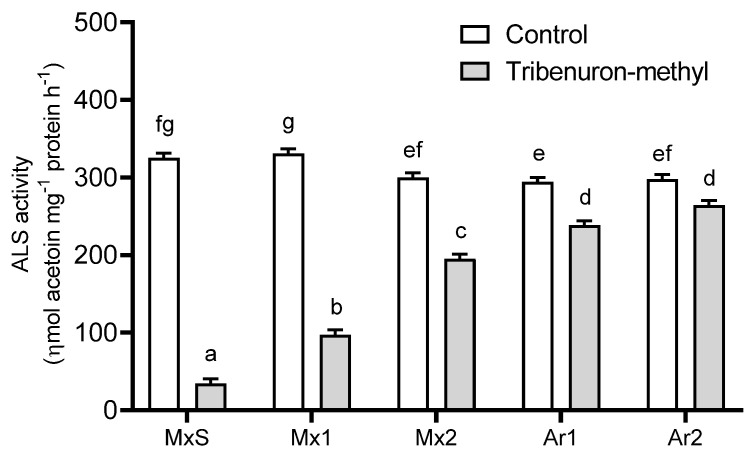
Acetolactate synthase (ALS) activity recorded in *Brassica rapa* populations without herbicide (control) and treated with tribenuron-methyl (10 g ai ha^−1^). Vertical bars represent the standard error of the mean (*n* = 5) and the letters above the bars indicate a statistical significance (*p* < 0.05).

TM (10 g ia ha^−1^) affected the ALS activity of the *B. rapa* populations in comparison to their untreated counterparts. TM inhibited the enzyme activity in all populations; however, MxS plants were the most sensitive and the herbicide reduced the ALS activity by 90%. In contrast, TM inhibited the ALS activity of the Argentinean populations (Ar1 and Ar2) between 9 and 19 % with no differences between them. These were the least sensitive populations to this herbicide. The ALS activity of the Mx1 and Mx2 populations differed from the MxS and Argentinean ones. Mx1 was more sensitive to TM than Mx2, and the inhibition rate of herbicide-associated ALS activity was 70% and 35%, respectively (Figure 1).

### 2.4. Ethylene Production in Response to 2,4-D

**Figure 2:**
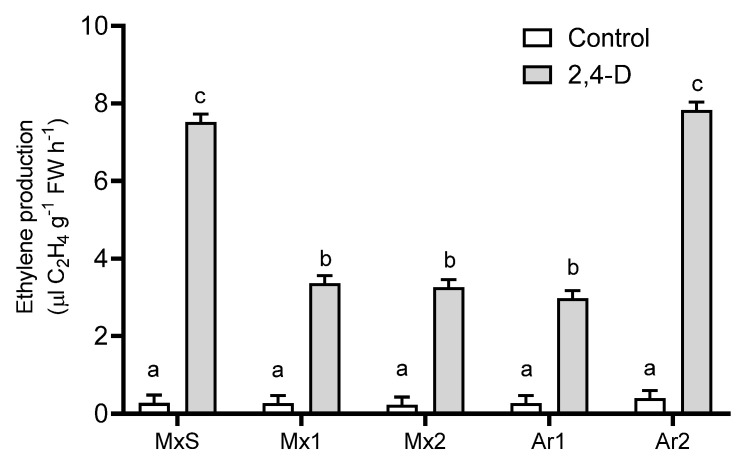
Ethylene production in *Brassica rapa* populations without herbicide (control) and treated with 2,4-D (500 g ai ha^−1^). Vertical bars represent the standard error of the mean (*n* = 5) and the letters above the bars indicate a statistical significance (*p* < 0.05).

In all *B. rapa* plants in comparison to their untreated counterparts, 2,4-D (500 g ai ha^−1^) stimulated ethylene production. In addition, there were differences in ethylene production between resistant populations. The Ar2 and MxS populations synthesized two-fold higher levels of ethylene than the Mx1, Mx2, and Ar1 populations (Figure 2).

### 2.5. Shikimic Acid Accumulation Assay

**Figure 3:**
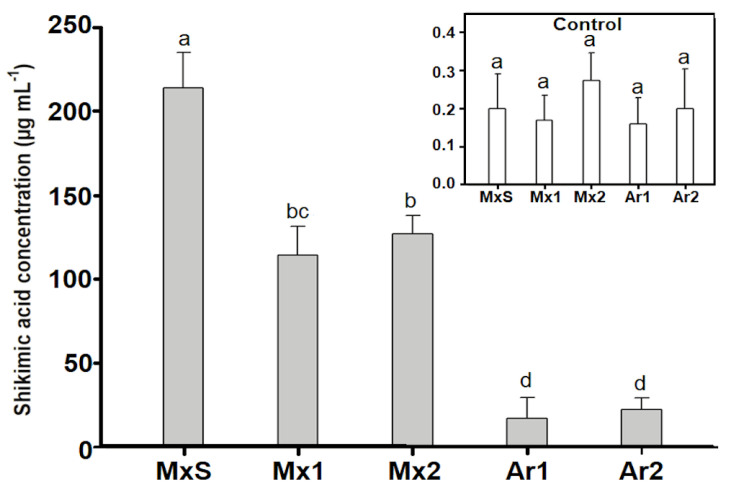
Shikimic acid concentration (µg/mL) in *Brassica rapa* populations without herbicide (control) and treated with glyphosate (600 g ae ha^−1^). Vertical bars represent the standard error of the mean (*n* = 5) and the letters above the bars indicate a statistical significance (*p* < 0.05).

Untreated control *B. rapa* plants presented paltry amounts of shikimic acid (>0.5 µg mL^−1^). However, after glyphosate treatment (600 g ha^−1^), the Argentinean and Mexican populations accumulated large amounts of shikimic acid. The lowest accumulation was recorded in the Ar1 and Ar2 populations, and the highest was recorded in MxS, which accumulated up to 20 times more shikimic acid than the Argentinean populations. The Mx1 and Mx2 populations accumulated close to half of the shikimate recorded for the MxS population (Figure 3).

## 3. Discussion

Evolutionary forces act on the genetic diversity in weed populations and explain how some plants survive herbicide treatment [14]. The current results demonstrated that *B. rapa* populations from different origins evolved in response to herbicides with MoA frequently used prior to planting or once the weeds have already emerged among wheat or barley crops. Glyphosate is widely used in reduced tillage cropping systems to control weeds during the fallow period before winter cereal is sown. Both Mexican and Argentinean *B. rapa* resistant populations showed resistance to glyphosate, but Mx1 and Mx2 were more sensitive to this herbicide than Ar1 and Ar2. In Argentina, the high level of glyphosate resistance (RF > 20) has been associated with a gene flow and introgression between populations of transgenic glyphosate-resistant *B. napus* and wild *B. rapa* [17]. In Mx1 and Mx2 populations, 70% of the plants survived the field dose of glyphosate, presenting a lower sensitivity compared to the Argentinean *B. rapa* populations, which suggests that different mechanisms of resistance would be involved. Interestingly, some herbicides, such as glufosinate, could be considered an alternative active principle to control glyphosate resistant Mx1, Mx2, Ar1, and Ar2 populations in fallow fields.

Undoubtedly, when *B. rapa* plants escape the glyphosate treatment, the pressure of control increases on herbicides registered for use on wheat or barley fields [22,23,24]. Resistant *B. rapa* populations from Argentina and Mexico presented cross-resistance to ALS-inhibitors since none of the plants were killed with herbicides from different chemical families (sulfonylureas, imidazolinones, and triazolopyridines) using this MoA. However, a phenotypic variability in response to TM was detected among populations. Cross-resistance to ALS-inhibiting herbicides has often been associated with target site mechanisms arising from mutant ALS genes, and these allelic variants have shown a different sensitivity to these herbicides [25]. From the 1980s onwards, this MoA has been massively used in wheat and barley fields in Argentina and Mexico [26,27]. Despite the usefulness of ALS inhibitors, the evolution of resistance to these herbicides is globally the most reported [4]. In Argentina, a gene transmission from an imidazolinone-resistant *B. napus* cultivar to *B. rapa* would be the origin of these herbicide-resistant populations [16]. In Mexico, the evolution of ALS-inhibitor herbicide resistance in *B. rapa* populations is novel, but a similar process of hybridization could be speculated. *Brassica napus* is an allopolyploid crop obtained though crosses between *B. rapa* and *B. oleracea* [28]; therefore, the hybridization of *B. napus* and *B. rapa* is very likely [29,30], even when the napus oilseed in Tlaxcala, Mexico, is not very extensive [21,31].

The herbicide-resistant weed challenge increases when the populations evolve resistance to herbicides with different MoAs [12]. Auxin-mimicking herbicides remain one of the groups of herbicides least prone to the evolution of resistance [4,32]. However, the resistance to auxin mimics was evidenced in Mx1, Mx2, and Ar1 *B. rapa* populations. In general terms, Brassicaceae species show a high sensitivity to phenoxy carboxylic acid herbicides, such as 2,4-D and MCPA, and these active principles have been used for over half a century in Argentina and Mexico [5,27]. The higher resistance to auxin mimics observed in the Mx1 and Mx2 populations may be associated with the widespread use of 2,4-D and the mixture of 2,4-D + dicamba for the control of leafy weeds in the High Valleys of Mexico (above 2000 masl), including the state of Tlaxcala, especially if the rainy season does not have a good start in early summer. Mexican farmers prefer auxin mimics to more expensive herbicides that control broadleaf and grass weeds since they often allow wild oats and other grasses to grow freely for later fodder (Dominguez-Valenzuela, personal observations). Variability in the response to 2,4-D and MCPA was detected at an intrapopulation level in Mx2, and these herbicides were effective to control Ar2 plants. However, all plants of Mx1 and Ar1 populations showed resistance to 2,4-D and MCPA. The mechanisms of resistance to synthetic auxin herbicides are unclear in most globally reported cases [5,32]. The 2,4-D resistant Mx1 and Ar1 populations showed 100% plant survival at a recommended dose of 2,4-D. Both populations presented low ethylene production induced by 2,4-D and their GR_50_ values were similar. However, the LD_50_ and RF values for Ar1 were approximately twice those for the Mx1 population. Cross-resistance to MCPA, 2,4-D, and fluroxypyr was reported in a *B. rapa* biotype from Argentina, but the mechanism of resistance is still unknown [33]. Moreover, Tafoya-Razo [34] mentioned that *B. rapa* evolved multiple-resistance to 2,4-D and ALS inhibitors in 2022, but he did not show the data; therefore, there are no official records of resistance to auxin mimetics of *B. rapa* from Mexico.

## 4. Conclusions

The current study reports on the evolution of cross- and multiple-resistance to glyphosate, ALS-inhibitors, and auxinic herbicides in *B. rapa* populations. A convergent phenotypic adaptation to herbicides commonly used in fallow and wheat/barley fields was detected in Mexican and Argentinean *B. rapa* populations. This convergent phenotypic evolution of cross- and multiple-resistance to herbicides can be explained by the widespread pattern of herbicide use that imposes a common selection pressure on the North and South American populations of *B. rapa*. These cases highlight the growing challenge of the resistance to more than one chemically unrelated herbicide for current and future weed management. Herbicides from other MoAs belonging to the PSI, PSII, GS, and HPPD inhibitors can be excellent tools for managing resistant populations of *B. rapa* in Mexico and Argentina.

## 5. Materials and Methods

### 5.1. Plant Materials

*Brassica rapa* seeds were collected from wheat fields in Argentina (provided by Ing. Agr. Ramón Gigón) and from barley fields in the state of Tlaxcala, Mexico (provided by Dr. Hugo E. Cruz-Hipolito) in 2021 (Table 3). The seeds were germinated in Petri dishes containing filter paper moistened with distilled water. The Petri dishes were placed in a growth chamber at 28/18 °C (day/night) with a photoperiod of 16 h, 350 μmol m^−2^ s^−1^ photosynthetic photon flux, and 80% relative humidity. All seedlings were transplanted into 250 cm^3^ pots (one plant per pot) containing 1:1 (*v*/*v*) sand/peat, placed in a greenhouse with a 16 h photoperiod, and watered daily until the herbicide treatments began.

### 5.2. Herbicide-Sensitivity Experiment

A greenhouse rapid resistance screening was carried out to ascertain whether these different *B. rapa* populations could survive the field dose application of herbicides with different mechanisms of action (MoAs) (Table 4). Herbicides were applied to plants that had four fully expanded leaves in a treatment chamber (Devries Manufacturing, Hollandale, Minnesota) equipped with a TeeJet 8002EVS flat fan nozzle that was calibrated to deliver 250 L ha^−1^ at 200 kPa at a height of 50 cm above the target. The experiment was arranged in a completely randomized design using 10 plants (one plant/pot) per treatment. Four weeks after application (WAA), the fresh weight and the number of surviving plants were determined. The surviving resistant individuals (>80% survivors) were grown to maturity, bulked, and allowed to produce seeds. The experiments were conducted twice at different times.

### 5.3. Dose–Response Experiments

Herbicide-resistant and susceptible plants were treated with 2,4-D, glyphosate, and tribenuron-methyl (TM) under the same conditions as in the previous experiment. The applied doses of each herbicide are detailed in Table 5. The experiment was arranged in a completely randomized design with five plants per treatment. The plants were grown in the greenhouse at 28/18 °C day/night and watered as necessary. At 4 WAA, the number of dead plants was recorded, and they were cut at ground level, stored individually in paper bags, dried at 60 °C for 4 days, and weighed. The data concerning the dry weight and plant mortality were transformed into a percentage relative to the untreated control to estimate the LD_50_ (herbicide dose required to kill by 50% of a weed population) and GR_50_ (dose required to reduce the shoot weight by 50% relative to the control) values.

### 5.4. ALS Enzyme Activity in Response to TM

TM doses (0 and 10 g ai ha^−1^) were applied to plants with four fully expanded leaves, as in the dose–response experiments. At 48 h after treatment (HAT), 3 g of leaf tissue was used to obtain the crude extract of the ALS enzyme, which was used to measure the enzymatic activity [35]. Acetoin absorbance obtained from acetolactate decarboxylation was measured using spectrophotometry (Beckman DU-640, Fullerton, CA, USA) at 520 nm. ALS activity (nmol acetoin mg^−1^ protein h^−1^) was calculated in TM-treated and non-treated plants. A completely randomized design with five replications per herbicide dose and population was used, and the experiment was replicated twice.

### 5.5. Ethylene Production in Response to 2,4-D

*Brassica rapa* plants with four fully expanded leaves were treated with 2,4-D solutions (0 and 500 g ai ha^−1^). Four hundred g of fresh shoot samples were taken at 24 HAT and placed in a 10 mL syringe with 1 mL of distilled water and then sealed [36]. The syringes were placed in a dark incubator at 27 °C for 4 h and 1 mL of the headspace gas was analyzed for ethylene (C_2_H_4_) using gas chromatography [37]. The C_2_H_4_ was expressed in nanoliter per gram of fresh weight per hour (nL g^−1^ fresh weight h^−1^). The experiment was arranged in a completely randomized design with five replicates and it was conducted twice.

### 5.6. Shikimic Acid Accumulation in Response to Glyphosate

Shikimic acid accumulation was determined in plants with four leaves and treated with glyphosate (0 and 600 g ae ha^−1^). Young leaf tissue samples (50 mg in 4 mm leaf discs) were taken at 48 HAT and placed in 2 mL tubes containing 1 mL of monoammonium phosphate (10 mM, pH 4.4 NH_4_H_2_PO_4_). The samples were incubated for 24 h under fluorescent light and after this time, the samples were frozen until they were analyzed [38]. For the analysis, the frozen samples were incubated at 60 °C for 30 min; then, 250 µL of hydrochloric acid (1.25 N) was added and the samples were again incubated at 60 °C for 15 min. Aliquots of 250 µL were transferred to new 1.5 mL tubes; then, a 500 µL solution of periodic acid (0.25% *w*/*v*) and sodium metaperiodate (0.25 % *w*/*v*) [1:1 (*v*/*v*)] was added. The samples were incubated at 25 °C for 90 min. Next, a 500 µL solution of sodium hydroxide (0.6 N NaOH) and sodium sulfite (0.22 N Na_2_SO_3_) [1:1 (*v*/*v*)] was added and mixed. The absorbance was measured using a spectrophotometer mod. DU-640 (Beckman Instruments Inc., Fullerton, CA, USA) at 380 nm. The results were expressed as micrograms of shikimate per milliliter HCL solution (µg shikimate mL^−1^) using a calibration curve with known concentrations of shikimate (3a,4a,5b-trihydroxy-1-cyclohexene-1-carboxylic acid, 99%, Sigma Aldrich, Inc., St. Louis, MI, USA). The experiment was conducted in a completely randomized design with five tissue samples from each population and glyphosate dose. The experiment was replicated twice.

### 5.7. Statistical Analysis

The data of the dose–response experiments were used to build non-linear log-logistic regression models with three parameters [39]. GR_50_, LD_50_ values, and resistance factors (RF = R-to-S ratios) were calculated, and the accuracy of the models was analyzed [25].

ANOVAs were performed to assess the differences between the ethylene production, shikimic acid accumulation, and ALS activity in the *B. rapa* populations. When necessary (*p* < 0.05), the means were distinguished using the Tukey test (Statistica^®^ v7.1. Statsoft, Inc., Hamburg, Germany, Europe).

## Figures and Tables

**Table 1:** Fresh weight (Fw) and plant survival percentages (%Sur) of *Brassica rapa* populations after herbicide treatments at field doses.

	Population	MxS		Mx1		Mx2		Ar1		Ar2	
Herbicide		Fw (g)	%Sur	Fw (g)	%Sur	Fw (g)	%Sur	Fw (g)	%Sur	Fw (g)	%Sur
Control		9.4 ± 1.6	100	10.1 ± 1.3	100	9.7 ± 2.6	100	9.4 ± 0.5	100	7.6 ± 1.1	100
Tribenuron-methyl		0.0	0.0	10.0 ± 3.0	100	10.0 ± 2.4	100	7.1 ± 0.9	100	8.0 ± 1.6	100
Florasulam		0.0	0.0	9.3 ± 0.7	100	10.7 ± 0.7	100	6.8 ± 0.9	100	7.1 ± 2.1	100
Imazamox		0.0	0.0	10.4 ± 0.6	100	10.6 ± 0.6	100	5.4 ± 1.3	100	4.9 ± 0.5	100
Iodo. + Meso. ^1^		0.0	0.0	5.4 ± 1.1	100	6.1 ± 2.4	100	4.3 ± 0.6	100	3.8 ± 0.6	100
2,4-D		0.0	0.0	7.5 ± 2.6	100	6.7 ± 3.7	80	4.7 ± 0.5	100	0.0	0.0
Clopyralid		7.8 ± 0.7	100	8.4 ± 1.7	100	5.9 ± 1.9	100	7.8 ± 1.8	100	7.2 ± 1.9	100
Dicamba		6.1 ± 0.9	100	10.7 ± 1.4	100	8.1 ± 2.7	100	6.1 ± 1.8	100	6.4 ± 0.6	100
Fluroxypyr		0.0	0.0	7.9 ± 1.4	100	7.1 ± 1.2	100	3.3 ± 0.2	100	0.0	0.0
MCPA		0.0	0.0	6.2 ± 2.5	100	3.8 ± 2.7	90	4.0 ± 0.4	100	0.0	0.0
Glyphosate		0.0	0.0	3.8 ± 0.4	70	4.2 ± 0.5	70	10.0 ± 1.2	100	8.1 ± 0.7	100
Atrazine		0.0	0.0	0.0	0.0	0.0	0.0	0.0	0.0	0.0	0.0
Bromoxynil		0.0	0.0	0.0	0.0	0.0	0.0	0.0	0.0	0.0	0.0
Glufosinate		0.0	0.0	0.0	0.0	0.0	0.0	0.0	0.0	0.0	0.0
Mesotrione		0.0	0.0	0.0	0.0	0.0	0.0	0.0	0.0	0.0	0.0

^1^ Iodo. + Meso. = iodosulfuron-methyl sodium + mesosulfuron-methyl.

**Table 2:** Parameters of the non-linear log-logistic model ^1^ used to estimate the necessary dose (g ai ha^−1^) of tribenuron-methyl, 2,4-D, and glyphosate in order to reduce the dry weight (GR_50_) and plant survival (LD_50_) by 50% in *Brassica rapa* populations.

Herbicide	Population	Dry Weight Reduction				% Plant Survival			
		d	b	GR_50_ (95% CI)	RF	d	b	LD_50_ (95% CI)	RF
Tribenuron-methyl	MxS	100.2	2.15	0.28 (0.25–0.31)	--	100.1	7.7	0.54 (0.43–0.66)	--
	Mx1	100.2	1.3	74.4 (56.9–92.9)	266	100.7	3.4	511 (430–593)	947
	Mx2	104.2	1.3	259 (204–314)	925	100.1	3.4	1535 (1294–1775)	2842
	Ar1	100.6	1.1	405 (296–513)	1445	100.9	2.7	1434 (1182–1687)	2656
	Ar2	99.9	1.4	664 (530–797)	2371	100.1	4.0	2197 (1875–2519)	4069
2,4-D	MxS	99.6	2.7	43.3 (39.4–47.2)	--	100.5	3.7	117 (106–128)	--
	Mx1	96.2	0.96	398 (335–461)	9.2	99.7	3.3	584 (553–614)	5.0
	Mx2	94.5	0.96	385 (314–456)	8.9	100.1	94	675 (608–743)	5.8
	Ar1	99.0	1.2	337 (263–411)	7.8	100.1	4.0	1099 (962–1235)	9.4
	Ar2	99.7	1.5	48.7 (38.8–58.5)	1.1	101.2	3.4	182 (158–207)	1.5
Glyphsate ^2^	MxS	100.1	0.7	97 (76–118)	--	100.7	2.9	225 (214–236)	--
	Mx1	98.5	1.7	355 (314–395	3.7	100.0	14.6	793 (643–943)	3.5
	Mx2	98.3	2.3	329 (301–357)	3.4	100.0	10.8	600 (457–743)	2.7
	Ar1	99.6	1.4	5153 (4270–6036)	53.0	100.3	2.9	9505 (8471–10,540)	42.3
	Ar2	99.2	1.9	2351 (2016–2687)	24.2	100.5	2.2	6849 (5995–7703)	30.5

^1^ Y = *d*/(1 + *b*(*log* (*x*) − *log* (*e*))); where *d* is the upper limit, *b* is the slope at the inflection point of the curve, *e* is the plant response at 50% (GR_50_ or LD_50_), and *x* is the herbicide dose. Resistance factor (RF) = R/S. ^2^ g acid equivalent (ae) ha^−1^.

**Table 3:** Features of *Brassica rapa* populations from Argentina (Arg.) and Mexico (Mex.) used in this research.

Population	Location, Country	Crops	Herbicide History	Coordinates
Ar1	Tandil, Arg.	Fallow and wheat	Glyphosate, metsulfuron-methyl, 2,4-D	37°13′22.8″ S 59°17′42.0″ W
Ar2	San Cayetano, Arg.	Fallow and wheat	Glyphosate, imazamox, 2,4-D	38°17′42.0″ S 59°23′09.6″ W
MxS	Texcoco, Mex.	No crops	Non treated	19°29′34.8″ N 98°52′30.0″ W
Mx1	Tlaxcala, Mex.	Barley	Glyphosate, iodosulfuron-methyl sodium + mesosulfuron-methyl, 2,4-D amine	19°36′14.4″ N 98°09′57.6″ W
Mx2	Tlaxcala, Mex.	Barley	Glyphosate, iodosulfuron-methyl sodium + mesosulfuron-methyl, 2,4-D amine	19°32′16.8″ N 98°09′57.6″ W

**Table 4:** Mechanism of action (MoA), active ingredient (ai), manufacturer, commercial product, and rate (g ai ha^−1^) of the herbicide treatments applied at field doses on *Brassica rapa* populations from Argentina and Mexico.

MoA/HRAC Group	Herbicide	Manufacturer	Commercial Product	Rate
ALS inhibitor/2	Tribenuron-methyl	Nufarm	75% *w*/*v*, Primma^®^ SL	20
	Florasulam	Nufarm	5% *w*/*v*, Fragma^®^ SL	5
	Imazamox	BASF	4% *w*/*v*, Pulsar^®^ 40 SC	40
	Iodosulfuron + Mesosulfuron	Bayer CropScience	5% + 0.75% *w*/*w*/*v*, Hussar^®^ Plus	150 ^1^
Auxin mimics/4	2,4-D	Nufarm	60% *w*/*v*, U 46 D Complet^®^ SL	600
	Clopyralid	Corteva	72% *w*/*v*, Lontrel^®^ WG	300
	Dicamba	Syngenta	48% *w*/*v*, Banvel^®^ D WG	150
	MCPA	Nufarm	40% *w*/*v*, Procer M-40^®^	800
	Fluroxypyr	Nufarm	20% *w*/*v*, Praxis^®^ EC	200
EPSPS inhibitor/9	Glyphosate	Bayer CropScience	36% *w*/*v*, Roundup^®^ SL	960 ^2^
PSII inhibitor/5	Atrazine	Syngenta	90% *w*/*v*, Gesaprim^®^ SL	1550
PSII inhibitor/6	Bromoxynil	Nufarm	38.5% *w*/*v*, Emblem Flo^®^ SC	385
GS inhibitor/10	Glufosinate	BASF	15% *w*/*v*, Finale^®^ SL	600
HPPD inhibitor/27	Mesotrione	Syngenta	48% *w*/*v*, Callisto^®^ SC	150

MoA: ALS–acetolactate synthase, EPSPS–5-enolpyruvylshikimate-3-phosphate, PSI–photosystem I, PSII, photosystem II, GS–glutamine synthetase, HPPD–4-hydroxyphenylpyruvate dioxygenase. HRAC: herbicide resistance action committee. ^1^ mL of the commercial product plus 500 mL of Biopower^®^ (27.65% *w*/*v*, sodium alkyl ether sulfate); ^2^ g acid equivalent (ae) ha^−1^.

**Table 5:** Herbicide doses applied to susceptible and resistant *Brassica rapa* populations in dose–response assays to estimate the GR_50_ and LD_50_ values.

Herbicide	Dose (g ai ha^−1^)
2,4-D	Susceptible: 0, 40, 80, 160, 320, 640, and 1280 Resistant: 0, 40, 80, 160, 320, 640, and 1280
Glyphosate ^1^	Susceptible: 0, 10, 20, 40, 80, 160, 320, and 640 Resistant: 0, 80, 160, 320, 640, 1280, 2560, 5120, and 10,240
Tribenuron-methyl	Susceptible: 0, 0.125, 0.25, 0.5, 1, 2, 4, and 10 Resistant: 0, 20, 40, 80, 160, 320, 640, 1280, and 2560

^1^ g acid equivalent (ae) ha^−1^.

## Data Availability

Data sharing is not applicable.
